# Imelda Lee—A memorial note

**DOI:** 10.1111/1753-0407.13606

**Published:** 2024-07-29

**Authors:** Zachary Bloomgarden

**Affiliations:** ^1^ Department of Medicine, Division of Endocrinology Diabetes and Bone Disease Icahn School of Medicine New York USA

Imelda Lee was one of the most important members of the *Journal of Diabetes* team—our founding administrator. Imelda started the *Journal of Diabetes* group with all of us more than 15 years ago. We pay attention to the scientists and physicians who nominally “run” the Journal, but it would be wrong to overlook those crucial to the daily operations “behind‐the‐scenes.”

Imelda grew up in Hong Kong in the early 1950s, and her sisters Lee and Frances remembered her to us as a fierce protector of all her family throughout her youth. After her secondary education, she worked in Hong Kong, then moved to the United States, and while working with her family at their business in Indiana, she was also able to attend and graduate from Purdue University, much to the admiration of those who knew her. She returned to China and moved to Shanghai, where she became an administrator at Rui Jin Hospital. She was recruited to manage the *Journal of Diabetes* as it was being started, when one of us (Z.B.) had the pleasure of meeting her. The same fierce spirit that led to her defending her siblings in Hong Kong and to her both working and going to college at night in the United States, led to her wonderful work at the new Journal. The impact of her administrative work was felt by all of us and by the editorial group; being both in China and in the United States, she was able to literally keep us going “24 × 7.” She challenged all of us to work harder, to learn the quirks of the Journal's complex computer system, and to reach out to the world of diabetes research to help develop the Journal in its formative period. She was quiet unless there was an issue, but then she would be fierce in helping all of us to accomplish more.

The Biblical term *Eshet Chayil* is usually translated as “Woman of Valor,” but has multiple meanings, referring as well to capacity and power, and Imelda was indeed a woman of true inner strength who exemplified wisdom and industry in her professional endeavors. Above all, she was guided by a strong moral compass, following a basic set of principles, accepting our efforts and uplifting and empowering us, and leaving us better for being her colleagues. Her active and optimistic attitude toward difficulties also greatly influenced us, with her readiness to help others a model for all to emulate. Imelda's deep faith was always an inspiring example to us but never more so than when she became ill several years ago. We did not hear words of unhappiness or despair; rather, she exemplified courage in the face of adversity.

We remember her, and we deeply miss her. As much as any of us, Imelda truly was a founder of the Journal, and we hope that the concept of the *Journal of Diabetes* in supporting a dialog between East and West in all aspects of the understanding of diabetes will continue as a memorial to her efforts.

